# Screening for cardiovascular disease using age alone: reflections on a paper peer-reviewed as both ‘radical’ and ‘unsurprising’

**DOI:** 10.1258/jms.2011.011093

**Published:** 2011-09

**Authors:** Richard Smith

**Affiliations:** Director, UnitedHealth Chronic Disease Initiative; Former Editor, *BMJ*

Using simply age to screen for cardiovascular disease is as effective as more complicated methods using blood pressure and serum cholesterol. That is the main conclusion of a study published in *PloS One* in May by Nick Wald, Mark Simmonds and Joan Morris.^1^ I consider here whether the message is right, what the implications might be and what we might learn from the prolonged passage to publication of this paper.

The authors used a Monte Carlo simulation with 500,000 people aged 0–89 to reach their conclusion. Taking being 55 or over as a positive test will detect 86% of cardiovascular events with a 24% false-positive rate. This simple assessment is compared with screening everybody from age 40 at five-yearly intervals using the standard Framingham risk score until people reach the risk of a 20% chance of a cardiovascular event in the next 10 years, the cut-off for treatment recommended by the National Institute of Health and Clinical Excellence (NICE). For the same 86% detection rate the false-positive rate is 21%. In other words, the two methods are effectively the same; almost nothing is gained from a series of visits to doctors, measurements, and blood tests.

Can this be right? It is. I write this after reading the comments of 24 reviewers of the paper. None of them seriously disputes the conclusion. Indeed, many say that the finding is unsurprising because, within the Framingham score of risk, age is so dominant.

The finding is, however, counterintuitive and contrary to current perception. Can people's family history, smoking status, blood pressure, serum lipids, and weight – all recognized risk factors for cardiovascular disease – not make much difference? As the paper says, ‘causal CVD risk factors, even in combination, are poor CVD screening tests.’^1^ Risk factors and screening tests are different. Many will also wonder why it is that we have a whole industry of screening tests – not only Framingham but also the Reynolds risk score^2^ or QRISK2^3^ – if age alone is just as good. There is also substantial research effort being applied to using genetic and other biomarkers to try and predict more accurately who will have heart attacks and strokes, research that has had disappointing results.^4^

Should we then abandon screening people for risk of cardiovascular events using the various scores and use simply age? People wouldn't have to visit doctors for screening assessments. They wouldn't have to have blood tests. They wouldn't have to try to understand what their Framingham score meant, and they wouldn't be divided into healthy sheep and unhealthy goats. Complicated risk assessments might end, but risk reduction – encouraging and helping people to stop smoking, lose weight, increase physical activity, eat healthier diets, and drink less alcohol – should continue. Others apart from doctors and nurses can do this work.

Risk reduction is sensible for everybody, but the point of risk assessment is to limit treatment to those above a specified risk. It is well recognized now that it is a person's overall risk (so-called ‘global risk’) that should be assessed and not simply raised blood pressure or serum lipids. One implication of the new study is that everybody might begin treatment at 55. This fits with the strategy proposed by Wald and Law in 2000^5^ and in 2003 in the *BMJ*^6^ to take a polypill containing blood pressure lowering drugs, a statin, and possibly aspirin and folic acid. This remains a controversial idea, although less controversial than when first described. Several companies in India have manufactured polypills, and two trials have been published showing their effect on measures like blood pressure and serum lipids.^7,8^

So will complicated risk assessments be abandoned? Perhaps not in the short term as we know that there is a long lag between evidence and action and as there is too much vested interest in both conducting the assessments and trying to devise new ones. Both are industries with markets to protect. The new evidence, although not surprising, does strengthen the case for the strategy of offering the polypill to everybody at 55, but this strategy also threatens vested interests and traditional thinking. Pharmaceutical companies see lucrative markets being destroyed. Doctors, particularly cardiologists, are sensitive to the implicit criticism that their strategy of assessing risk and treating is unnecessarily complex and overlooks the fact that many cardiovascular events occur in people without high risk factors, the ‘prevention paradox’.^9^ Public health practitioners fear the polypill offers a licence to people to avoid healthy lifestyles, although there is every reason for people to combine the polypill with healthy lifestyles and no reason not to. Finally, some worry about medicalization, although, as I've argued elsewhere, giving people the pill without tests is the opposite of medicalization in that those who take the polypill can avoid falling into the hands of doctors.^10^

One of the aspects of this paper that fascinates me – as a former editor of the *BMJ* and a current member of the board of the Public Library of Science – is its publication history. A version of the paper was first submitted to a journal, the *BMJ*, in March 2009. It was finally published in *PloS One* in May 2011, more than two years after it was first submitted. During that time the paper has been rejected seven times by four journals, including *PloS One* at first, and reviewed by 24 reviewers. At a conservative estimate of two hours per review this is more than a week of academic time. If the academics are paid at a rate of £50 an hour, again conservative, the cost is over £2000. That figure does not include the editorial costs or the opportunity costs. The academics might have spent their time doing something much more valuable than reviewing a paper that 23 other reviewers had also reviewed.

This long delay and high cost might have been justified if what was eventually published was much superior to what was initially submitted. It's different, but the central message that age alone is as good as complex risk assessment scores is still the same and has not been seriously disputed. The comments of the reviewers could have been a useful discussion around the paper, part of the process of digesting it and deciding its true importance. As it is, their comments are lost in the memory stores of editorial computers. It is not clear to me whether the journals rejected the paper because it was too unsurprising or too radical in its threat to established interests or, paradoxically, both.

What is clear is that nothing would have been lost and much gained if this paper had been published straight away and the debate over its value had been conducted in public rather than behind closed doors for over two years at considerable expense. The evidence, as opposed to the opinion, on prepublication peer review shows that its effectiveness has not been demonstrated and that it is slow, expensive, largely a lottery, poor at spotting error, biased, anti-innovatory (as perhaps in this case), prone to abuse, and unable to detect fraud.^11^ The global cost of peer review is $1.9 billion^12^, and it is a faith-based rather than evidence-based process, which is hugely ironic when it is at the heart of science.

My conclusion is that we should scrap prepublication peer review and concentrate on postpublication peer review, which has always been the ‘real’ peer review in that it decides whether a study matters or not. By postpublication peer review I do not mean the few published comments made on papers, but rather the whole ‘market of ideas,’ which has many participants and processes and moves like an economic market to determine the value of a paper. Prepublication peer review simply obstructs this process – as happened with this important paper showing that age alone is enough for screening for cardiovascular disease.

**Declaration of interest:** RS was the editor of the *BMJ* and the chief executive of the BMJ Publishing Group, which once owned the *Journal of Medical Screening*, and is a member of the board of the Public Library of Science. He is also a long established enthusiast for the polypill and takes it every night.
